# Management of Patients With Chemotherapy-Induced Nausea and Vomiting

**Published:** 2017-04-01

**Authors:** Sally Yowell Barbour

**Affiliations:** Duke University Hospital, Durham, North Carolina

## Abstract

Despite advances in the management of chemotherapy-induced nausea and vomiting (CINV), clinical management remains a challenge and CINV continues to be a side effect that patients fear most. Advanced practitioners can play a major role in evidence-based interventions for prevention and management of these side effects of treatment.

Despite advances in the management of chemotherapy-induced nausea and vomiting (CINV), clinical management remains a challenge, said Sally Yowell Barbour, PharmD, BCOP, CPP, of Duke University Hospital in Durham, North Carolina, during a 2016 JADPRO Live presentation. "Nausea and vomiting is one of the side effects patients fear the most," said Dr. Barbour.

Studies of patient perceptions of the most severe side effects of cancer chemotherapy have consistently shown nausea and vomiting at or near the top of rankings of importance. In a study conducted more than 30 years ago, patients rated vomiting as the most troublesome side effect of chemotherapy, followed by nausea (Coates et al., 1983). A decade later, nausea topped the list, followed by tiredness, loss of hair, effects on the family, and vomiting (Griffin et al., 1996).

For most of the next decade, nausea remained the number-one concern among patients, whereas the importance of vomiting declined (de Boer-Dennert et al., 1997; Lindley et al., 1999). By 2004, fatigue had supplanted nausea as the most troubling side effect of chemotherapy, and vomiting had disappeared from the list altogether (Hofman et al., 2004).

"As we’ve had advancements in pharmacologic management of this side effect with the introduction of the serotonin (5-HT3) receptor antagonists, vomiting fell down in the rankings, but nausea was still an issue," said Dr. Barbour. "With the introduction of newer agents, such as the NK [neurokinin]-1 antagonists, we’ve learned better how to categorize the risk factors. Vomiting has continued to stay near the bottom of the top five, but nausea continues to be one of the main problems we face today in the management of patients."

## GUIDELINE DEVELOPMENT

Recognition of risk factors for CINV and emergence of improved management options have led to the development of national and international guidelines for prevention and treatment of CINV. Adherence to the guidelines and improved use of effective antiemetic agents have led to better control of CINV and resource utilization.

"Many practices have electronic medical records with chemotherapy regimens prebuilt that include supportive care," revealed Dr. Barbour. "We now have multiple classes of drugs to manage CINV."

Even so, a substantial proportion of patients treated with chemotherapy develop emesis, particularly during the delayed phase. The factors below contribute to the persisting challenge of managing CINV:

- Inconsistent application of guideline-based antiemetic regimens
- Nonadherence to antiemetic regimens, particularly regimens for delayed-phase emesis
- Affordability of medications
- Guidelines’ lack of direction for various scenarios and their tendency to focus on two principal issues: emetogenicity of single-dose chemotherapy and pattern of CINV (acute vs. delayed).

Additionally, insufficient data exist to provide guidance for a number of issues: incorporation of patient-specific risk factors for emesis; multiday chemotherapy; stem cell transplant; oral agents; and management of CINV in children.

Effective prevention and management of CINV begin with a careful assessment of an individual patient’s risk (Hesketh, 1999). The emetogenic potential of a chemotherapy regimen usually heads the list of factors for review. Younger patients (< 50 years) and women tend to be at increased risk for CINV (Hesketh, 1999). A history of low alcohol intake or abstinence, a history of motion sickness, and a history of emesis during pregnancy also raise the risk of a patient’s likelihood of developing CINV (Hesketh, 1999).

The American Society of Clinical Oncology (ASCO) and the National Comprehensive Cancer Network (NCCN) Clinical Practice Guidelines for managing CINV established four categories of emetogenic potential, defined by the observed frequency of emesis in patients treated with a regimen (Basch et al., 2011; NCCN, 2016). Highly emetogenic regimens cause emesis in more than 90% of treated patients in the absence of antiemetic therapy. Moderately emetogenic regimens induce CINV in 30% to 90% of patients. Low-risk regimens induce CINV in 10% to 30%, and regimens with minimal emetogenic potential cause nausea and/or vomiting in < 10% of these patients (NCCN, 2016). The NCCN Guideline includes dozens of chemotherapeutic agents, organized by emetogenic potential and route of administration (intravenous [Table 1] and oral [Table 2]).

**Table 1:**
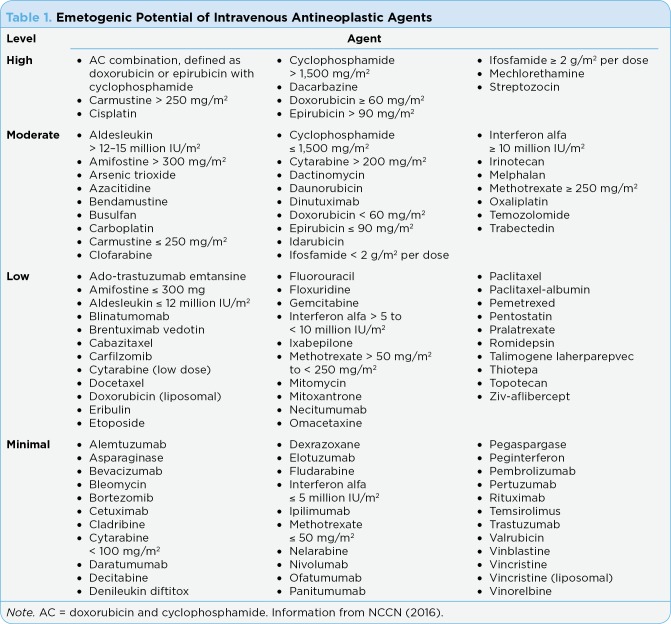
Emetogenic Potential of Intravenous Antineoplastic Agents

**Table 2:**
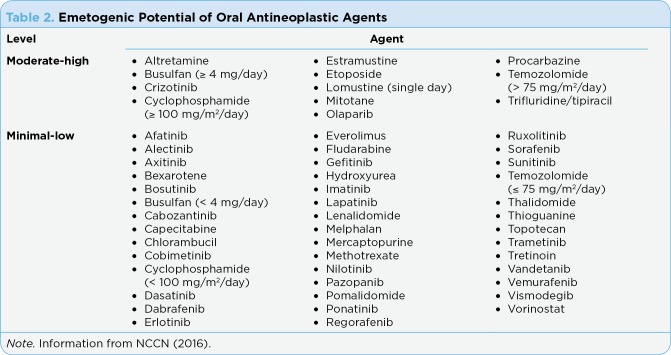
Emetogenic Potential of Oral Antineoplastic Agents

"The two that stand out the most, both of which are widely used, are cisplatin and the AC (doxorubicin/cyclophosphamide) combination," said Dr. Barbour. "Any dose of cisplatin is classified as highly emetogenic. The AC combination once was classified as moderately emetogenic, but all the guidelines now treat this combination as highly emetogenic. Any patient treated with this combination should receive all of the drugs used for highly emetogenic chemotherapy."

Other agents that have received considerable attention in updates to clinical guidelines include carboplatin, irinotecan, lower-dose anthracyclines, and oxaliplatin.

Until fairly recently, clinical guidelines for CINV focused primarily on intravenous drugs. Increased availability of oral chemotherapeutic agents has led to some reassessment of CINV guidance. Oral agents tend to be well tolerated, and most of them fall into the low- and minimal-emetogenic categories, said Dr. Barbour. Notable exceptions, however, include oral cyclophosphamide and procarbazine.

Recently, ASCO, NCCN, the Multinational Association for Supportive Care in Cancer (MASCC), and the European Society for Medical Oncology (ESMO) updated their clinical guidance for managing CINV (Hesketh et al., 2016; NCCN, 2016; MASCC, 2016). Collectively, the organizations made consistent recommendations.

All of the guidelines recommend that patients in the high-risk category receive the three-drug combination of a 5-HT3 antagonist, dexamethasone, and an NK-1 antagonist. The guidelines also include AC chemotherapy in the high-risk or special-risk category requiring treatment with three antiemetics.

In a departure, the MASCC/ESMO guideline includes carboplatin in the high-risk category. Patients with a moderate risk for emesis should receive a 5-HT3 antagonist and dexamethasone. Patients in the low-risk category can be managed with a 5-HT3 antagonist, dexamethasone, or a dopamine antagonist. Routine prophylaxis is not required for patients with a minimal-emetogenic risk.

Patients who develop breakthrough CINV pose additional challenges. Several strategies have proven useful in this setting (NCCN, 2016; Navari et al., 2013), including an additional agent from a different therapeutic class, around-the-clock treatment as opposed to as-needed administration, and if the patient is vomiting, intravenous or rectally administered medication, she said.

For patients with breakthrough CINV, response to antiemetic medication during the acute and delayed phases prior to initiation of the second cycle of chemotherapy should be reassessed, Dr. Barbour advised. Consider whether an alternative regimen might be needed. An NK-1 receptor antagonist can be added to treatment if it has not previously been used. The antipsychotic olanzapine (Zyprexa) might also warrant consideration if not already tried.

## NEW THERAPEUTIC OPTIONS

The pharmacologic options for managing CINV have increased in recent years, including the approval of two new oral NK-1 antagonists: netupitant and rolapitant (Varubi).

Netupitant, which selectively blocks the substance P NK-1 receptor, is available in a fixed-dose combination with palonosetron (Akynzeo), and it has US Food and Drug Administration (FDA) approval for prevention of acute and delayed nausea and vomiting. The combination plus dexamethasone was evaluated in a phase III randomized clinical trial involving 1,455 patients receiving AC chemotherapy (Aapro et al., 2014). The patients were randomized to receive netupitant/palonosetron or oral palonosetron, and patients in both groups also received oral dexamethasone.

The trial had a primary endpoint of complete response during the delayed phase (25–120 hours), defined as no emesis and no use of rescue medication. The combination demonstrated superiority for the primary endpoint (77% vs. 70%, *p* = .001), as well as complete response in the acute phase (88% vs. 85%, *p* = .047) and overall (0–120 hours, 74% vs. 67%, *p* = .001).

"The most common treatment-related adverse events were headache and constipation," said Dr. Barbour. "This fixed-dose combination offers guideline-based prophylaxis with convenient, single-day treatment."

Rolapitant is an NK-1–selective competitive antagonist, approved for use in combination with other antiemetic agents to prevent delayed nausea and vomiting associated with initial and repeated courses of chemotherapy. The drug was evaluated in a randomized phase III trial involving 1,369 patients treated with moderately emetogenic chemotherapy (Schwartzberg et al., 2015). Patients received rolapitant in combination with granisetron and dexamethasone or placebo plus granisetron and dexamethasone.

The trial met the primary endpoint of improvement in complete response during the delayed phase (71.3% vs. 61.6%, *p* < .001). The rolapitant regimen was not superior during the acute phase, but it did demonstrate overall superiority (68.6% vs. 57.8%, *p* < .001).

In contrast to other NK-1 antagonists, rolapitant does not inhibit or induce the CYP3A4 metabolic pathway, Dr. Barbour noted. As a result, the agent has less potential for significant drug-drug interactions, and dexamethasone can be administered at the full dose (24 mg), whereas reduction to 12 mg is required for use with other NK-1 antagonists.

Another newer antiemetic option is not a new drug but rather a repurposed agent. The oral antipsychotic olanzapine affects multiple neurotransmitters, including alpha 1, dopamine, histamine H1, muscarine, and serotonin type 2 receptors. Several phase II/III trials demonstrated olanzapine’s antiemetic activity, but most of the studies had limitations, such as small sample size, inadequate blinding, and low statistical power, according to Dr. Barbour.

Recently, an olanzapine-containing antiemetic regimen was evaluated in a randomized phase III trial involving 400 patients receiving initial chemotherapy that included cisplatin or AC (Navari et al., 2016). Patients received one of two antiemetic combinations: olanzapine/aprepitant/5-HT3 receptor antagonist/dexamethasone or placebo plus the same three drugs included in the olanzapine combination.

The trial had a primary endpoint of complete absence of nausea. The olanzapine combination demonstrated superiority in the overall analysis (37% vs. 22%, *p* = .002) and in both the acute (74% vs. 45%, *p* < .001) and delayed (42% vs. 25%, *p* = .001) phases.

For the secondary endpoint of complete response (no emesis or use of rescue medication), the olanzapine-containing combination proved superior overall control (64% vs. 41%, *p* < .001), including in the acute phase (86% vs. 65%, *p* < .001), and in the delayed phase (67% vs. 52%, *p* = .007). Olanzapine was, however, associated with more sedation, a known side effect of the drug (20% vs. 7% on day 2).

"Olanzapine demonstrated improvement in nausea control," said Dr. Barbour, "and offers an option for a regimen without an NK-1 antagonist."

## QUALITY-OF-LIFE ISSUES

Chemotherapy-induced nausea and vomiting can profoundly affect a patient’s quality of life. In a recent survey of 400 patients receiving chemotherapy, almost three-fourths said the adverse effects of chemotherapy made them want to avoid future cycles of therapy (Hematology/Oncology Pharmacy Association, 2016). More than half had to cancel personal plans; almost half had to change their eating habits; more than 40% avoided exercise or physical activity; almost 40% took time off from work; and 30% had a more negative outlook on their prognosis.

Data from the Anti-Nausea Chemotherapy Registry (ANCHOR) showed that nausea has a greater negative effect on patients than does vomiting (Bloechl-Daum, Deuson, Mavros, Hansen, & Herrsted, 2006). Additionally, patients reported that highly emetogenic chemotherapy had a greater negative impact on their lives than did moderately emetogenic chemotherapy.

## ROLE OF ADVANCED PRACTITIONERS

Advanced practitioners should take an active role in managing CINV, said Dr. Barbour. Opportunities to improve practice and patient experience include participating in development or implementation of institution-specific guidelines for managing CINV; ensuring adherence to guidelines; participating in the planning of patient therapy; educating patients and the oncology team about CINV; assessing patient risk factors; and creating medication-management protocols for CINV.

For example, Dr. Barbour shared the results of a study of a pharmacist-driven initiative to improve adherence to an institutional protocol for CINV (Elshaboury & Green, 2011). The study involved 106 inpatients receiving emetogenic chemotherapy, 55 of whom participated in a pharmacist-driven protocol and 51, in a physician-driven protocol.

The data showed that 85% of patients received protocol-recommended care in the pharmacist group compared with 33% of patients in the physician group (*p* < .0001). Additionally, 20% of patients in the physician group received excessive CINV prophylaxis vs. 2% in the pharmacist group, and the number of breakthrough doses did not differ between the two groups.

"Chemotherapy-induced nausea and vomiting is still a significant problem for many patients, especially delayed CINV," said Dr. Barbour. "A therapeutic approach of combining antiemetics with different mechanisms gives the best results in preventing CINV. Advanced practice providers, nurses, and pharmacists should play key roles in helping to assess and manage CINV."
